# The Use of Thrombectomy during Primary Percutaneous Coronary Intervention: Resurrecting an Old Concept in Contemporary Practice

**DOI:** 10.3390/jcm13082291

**Published:** 2024-04-15

**Authors:** Zahir Satti, Muntaser Omari, Bilal Bawamia, Timothy Cartlidge, Mohaned Egred, Mohamed Farag, Mohammad Alkhalil

**Affiliations:** 1 Cardiothoracic Department, Freeman Hospital, Freeman Road, Newcastle upon Tyne NE7 7DN, UK; zahir.satti@nhs.net (Z.S.); muntaser.omari@nhs.net (M.O.); bilal-reshad.bawamia@nhs.net (B.B.); t.cartlidge@nhs.net (T.C.); m.egred@nhs.net (M.E.); mohamedfarag@nhs.net (M.F.); 2 Translational and Clinical Research Institute, Newcastle University, Newcastle upon Tyne NE1 7RU, UK

**Keywords:** myocardial infarction, primary percutaneous coronary intervention, myocardial reperfusion, thrombectomy, thrombus aspiration, distal embolization, microvascular obstruction, ST-elevation myocardial infarction, no-reflow and slow-reflow phenomena

## Abstract

Optimal myocardial reperfusion during primary percutaneous coronary intervention (pPCI) is increasingly recognized to be beyond restoring epicardial coronary flow. Both invasive and non-invasive tools have highlighted the limitation of using this metric, and more efforts are focused towards achieving optimal reperfusion at the level of the microcirculation. Recent data highlighted the close relationship between thrombus burden and impaired microcirculation in patients presenting with ST-segment elevation myocardial infarction (STEMI). Moreover, distal embolization was an independent predictor of mortality in patients with STEMI. Likewise, the development of no-reflow phenomenon has been directly linked with worse clinical outcomes. Adjunctive thrombus aspiration during pPCI is intuitively intended to remove atherothrombotic material to mitigate the risk of distal embolization and the no-reflow phenomenon (NRP). However, prior trials on the use of thrombectomy during pPCI did not support its routine use, with comparable clinical endpoints to patients who underwent PCI alone. This article aims to review the existing literature highlighting the limitation on the use of thrombectomy and provide future insights into trials investigating the role of thrombectomy in contemporary pPCI.

## 1. Introduction

Myocardial reperfusion with a restoration of normal epicardial coronary blood flow during primary percutaneous coronary intervention (pPCI) for patients presenting with ST-segment elevation myocardial infarction (STEMI) is fundamental to salvage myocardium and improve survival [1,2]. However, despite adequate epicardial coronary flow, a significant proportion of patients (up to 50%) fail to achieve optimal myocardial reperfusion and develop a phenomenon referred to as no reflow (NRP) [3,4,5]. This phenomenon is described as reduced coronary antegrade flow (less than Thrombolysis In Myocardial Infarction (TIMI) III grade flow) without visible obstruction or dissection of the infarct-related artery (IRA), which occurs as a result of microvascular obstruction (MVO) with a subsequent increase in myocardial tissue impedance, leading to poor epicardial antegrade TIMI flow [6,7,8].

NRP can be assessed invasively using myocardial blush grade (MBG) or index microcirculatory resistance (IMR) [4,9,10]. It can also be detected using a wide range of non-invasive tools such as myocardial contrast echocardiography (MCE), myocardial perfusion scintigraphy (MPS), positron emission tomography (PET), and cardiac magnetic resonance imaging (MRI), which remains the gold standard of identifying and assessing the status of the microcirculation [11,12,13,14,15,16]. Contrast-enhanced cardiac MRI allows the measurement of MVO and is the modality of choice for assessing infarct size. Infarct size is a strong predictor of future clinical events beyond left ventricular (LV) ejection fraction and end-systolic volume index (ESVI) [17,18]. In fact, MVO has been proposed to be a surrogate of death and re-admission with heart failure in patients presenting with STEMI [15,19]. Irrespective of the way to assess coronary microcirculation, clinical outcomes are related to its impaired status [19,20], notwithstanding that poor microcirculatory function was also associated with in-hospital major adverse events, persistent ST-segment elevation, impaired LV function, and large infarct size [4,21,22].

Adjunctive tools during pPCI have been used to mitigate this risk and to preserve coronary microcirculation, which include a thrombectomy catheter. The direct association between thrombus burden and clinical outcomes, as well as microcirculation, would support the concept of removing the clot during pPCI. However, contemporary clinical trials failed to support the use of thrombectomy in patients presenting with STEMI. In this review, we aim to examine existing evidence on the use of thrombectomy for acute myocardial infarction (AMI) and provide future insights into upcoming technologies that may address the concept of thrombectomy in the STEMI population.

## 2. The Detrimental Role of Thrombus Burden in STEMI

The primary pathophysiologic process leading to AMI is related to the acute erosion or rupture of a vulnerable atheromatous plaque with the formation of an occlusive thrombus of epicardial coronary flow [20,23]. Despite the restoration of coronary epicardial flow by opening the IRA with pPCI (>90% of patients), optimal myocardial reperfusion is not always guaranteed, owing to distal microembolisation, myocyte necrosis, and changes in the integrity of the microvasculature [24]. Previous studies have highlighted the direct relationship between coronary microcirculation and thrombus burden. A well-validated scoring system was developed to predict the status of coronary microcirculation. The ATI score includes age, thrombus burden, and pre-stent index microcirculatory resistance, which was highly predictive of impaired microcirculation, defined as IMR > 40, with an area under the curve of 0.87 [25]. Importantly, thrombus burden carried the largest weight in predicting high IMR post-stenting. Furthermore, patients without a very large thrombus burden (i.e., a thrombus score less than 5; see Table 1 for classification) were less likely to develop impaired microcirculation in this study. Moreover, the ATI scoring system was validated in predicting MVO and infarct size on cardiac MRI and may identify patients at higher risk of adverse long-term events [26,27]. Collectively, this highlights the central role of thrombus burden on the functional status of coronary microcirculation in patients presenting with STEMI.

In addition to microcirculation, previous studies have assessed the relationship between thrombus burden and clinical outcomes. A meta-analysis of an individual patient level of more than 18,000 patients from the large thrombus aspiration (TA) trials (TAPAS, TASTE, and TOTAL) showed that patients with large (TIMI thrombus grade ≥ 3) compared to small thrombus burden (<3) had significantly higher cardiovascular (3.1% vs. 2.2%, *p* = 0.02), but not all-cause, mortality at 30 days (3.1% vs. 2.4%, *p* = 0.07) [29]. This difference remained evident after one-year follow-up, and likewise, cardiovascular (4.5% vs. 3.2%, *p* = 0.006) but not all-cause mortality (5.3% vs. 4.4%, *p* = 0.08) was significantly higher in patients with a large thrombus score. Interestingly, when categorizing patients using a thrombus score cut-off of 4, all-cause mortality was higher compared to patients with a thrombus score of <4 at 30 days (3.3% vs. 2.4%, *p* = 0.01) but not at 1-year follow-up (5.4% vs. 4.6%, *p* = 0.08) [29]. Collectively, this supports the notion that thrombus burden is a risk marker in patients presenting with STEMI.

Mechanistically, the prognostic value of thrombus burden is beyond its role at the site of plaque rupture. Previous studies have also highlighted the detrimental role of distal embolization during pPCI. This may occur spontaneously or by the mechanical breakage of thrombi during pPCI and was estimated to affect 20–40% of STEMI cases. Thrombus score is known to be a predictor of angiographically visible distal embolisation [30]. Histopathological data revealed that distal embolization is mainly plaque fragments and partially organized thrombi which are unlikely to respond to anti-thrombotic medications [4,30,31]. The data also showed that a high thrombus burden was a significant predictor of distal embolization, with an almost 3-fold increase in mortality in the TOTAL trial [4,30,31]. In addition to mechanical plugging, the microembolisation of atherothrombotic debris may result in an inflammatory response and the recruitment of additional inflammatory cells, leading to further oedema and vasospasm that inevitably contribute to a reduction in coronary flow [24].

Overall, existing evidence highlights the adverse role of thrombus burden in patients with STEMI. Its role as a risk marker is well established; however, whether reducing the thrombus burden will translate into better clinical outcomes remains very controversial. The results of clinical outcomes from trials on using thrombus aspiration have not been consistent and will be discussed in the following section.

## 3. Concept of Thrombectomy/Thrombus Aspiration

The primary intention of thrombectomy or TA is to remove the clot that has developed at the site of acute plaque rupture. This improves flow down the infarcted artery and may achieve effective tissue reperfusion by preventing distal embolisation during pPCI [32,33]. It is apparent that thrombus burden, distal embolisation, and impairment of the microcirculation are correlated with poor patient clinical outcomes, including all-cause mortality and heart failure [5,7,19,30,34,35].

Early studies assessed the role of TA in improving myocardial reperfusion using surrogates of patients’ clinical outcomes. The prospective single-centre REMEDIA (Randomised Evaluation of the Effect of Mechanical Reduction of Distal Embolisation by Thrombus-Aspiration in Primary and Rescue Angioplasty) trial randomised 100 consecutive patients before coronary angiography into manual TA using a 6-French Diver CE catheter (Invatec, Brescia, Italy) or standard. The primary endpoints were post-procedural angiographic (MBG ≥ 2) and electrocardiographic (ECG) (ST-segment resolution (STR) ≥ 70%). The authors showed better myocardial reperfusion parameters using TA (46%) compared to PCI alone (24.5%) (odds ratio (OR) of 2.6 (95% confidence interval [CI], 1.1–6.2), *p* = 0.025) [36]. These results were reinforced by the findings from the EXPIRA (Thrombectomy With Export Catheter in Infarct-Related Artery During pPCI) study, which included 175 STEMI patients. It highlighted the benefits of TA in achieving MBG ≥ 2 (80% vs. 60%, *p* = 0.001) and STR ≥ 70% (64% vs. 39%, *p* = 0.001) compared to no TA [37]. The contrast-enhanced cardiac MRI sub-study of the EXPIRA study recruited 75 patients and showed a significant reduction in MVO and infarct size at 3 months with the use of TA [37].

Collectively, the evidence from the REMEDIA and the EXPIRA studies supports the potential beneficial role of TA as an adjunctive therapy to pPCI, although both studies had a small sample size. TAPAS (Thrombus Aspiration during Percutaneous Coronary Intervention in Acute Myocardial Infarction) was a prospective randomized controlled trial (RCT) which recruited a relatively large sample size of 1071 patients and used a 6-French Export Aspiration Catheter [38]. The use of TA resulted not only in better microvascular reperfusion (MBG 0 or 1, 17.1% vs. 26.3%, *p* < 0.001) but also in a cardiovascular mortality reduction at 1 year (3.6% vs. 6.7%, hazard ratio 1.93, 95% confidence interval 1.11–3.37, *p* = 0.02) [39]. A subsequent meta-analysis by De Luca et al. reported a significant reduction in 30-day mortality with adjunctive manual TA during pPCI for AMI patients (1.7 vs. 3.1%, OR 0.58; 95% CI (0.34–0.98), *p* = 0.01) [40,41]. Similarly, 6–12-month mortality benefits were reported in an updated meta-analysis of 18 trials (including TAPAS) [40,41]. Given the findings from these studies, this was reflected in previous guidelines in 2008/2009 of the European Society of Cardiology (ESC) and the American Society of Cardiology (ACC)/American Heart Association (AHA), which recommended TA as a class IIa recommendation for routine use during pPCI [42,43].

However, larger studies including TASTE (Thrombus Aspiration in ST-Elevation Myocardial Infarction in Scandinavia) and TOTAL (Thrombectomy with PCI vs. PCI Alone in patients with STEMI) did not support the use of routine TA in patients presenting with STEMI. TASTE was conducted in 2013 and included 7244 STEMI patients within the Swedish Coronary Angiography and Angioplasty Registry (SCAAR) to investigate the primary endpoint of all-cause mortality at 30 days [44,45]. It showed no significant difference in the primary outcome of 30-day mortality between the two groups (2.8% vs. 3.0%, hazard ratio (HR) 0.94, [95% CI, 0.72–1.22]). It also showed no difference in stent thrombosis (HR 0.57, [95% CI, 0.20–1.02]) or recurrent MI reduction (HR 0.61, [95% CI, 0.34–1.07]) with the use of TA [44], with similar results at 1-year follow-up [46]. More importantly, TOTAL was the largest (*n* = 10,732) multi-centre prospective RCT comparing routine upfront manual TA with pPCI versus pPCI alone in STEMI [47,48]. The TOTAL study showed no difference in the incidence of the primary endpoints of cardiovascular death, recurrent MI, cardiogenic shock, and New York Heart Association (NYHA IV) heart failure at 180 days with the routine use of TA (6.9% vs. 7.0%; *p* = 0.86) and CV mortality (3.1% vs. 3.5%; *p* = 0.34) [48]. Importantly, there was a signal of harm given the small but significant increased risk of stroke in the TA arm (30-day stroke rates: 0.7% vs. 0.3%; *p* = 0.02) [48]. At 1-year follow-up, the primary endpoint occurred in 8% of patients undergoing routine TA as well as PCI alone (HR 1.00 [95% CI, 0.87–1.15], *p* = 0.99) [49]. Similarly, 1-year cardiovascular death was 4% in both groups (HR 0.93 [95% CI, 0.76–1.14]; *p* = 0.48) [49]. Interestingly, the difference in stroke continues to increase past 1-year follow-up, challenging the causal relationship with the use of TA (1.2% vs. 0.7%, HR 1.66 [95% CI, 1.10–2.51]; *p* = 0.015) [49]. Similarly, the INFUSE-AMI (Intracoronary Abciximab and Aspiration Thrombectomy in Patients with Large Anterior Myocardial Infarction) trial assessed the role of TA in 452 STEMI patients with occluded proximal or mid-left anterior descending artery to discern any benefits in patients with large infarcts [50,51]. TA did not result in a reduction in infarct size at 30 days, as assessed by MRI [52]. Subsequently, the 2021 ACC/AHA guidelines for Coronary Artery Revascularization downgraded the class of recommendation for routine TA to class III (no benefit) with a level of evidence of A [53]. Likewise, the most recent (2023) ESC guidelines for the management of acute coronary syndromes did not recommend the use of routine TA (class IIIA recommendation) [2]. TA studies are summarized and presented in Table 2.

## 4. The Disconnect between Mechanistic and Clinical Evidence

Previous data highlighted thrombus burden as a risk marker in patients presenting with STEMI. Subsequently, small RCTs showed benefits in angiographic surrogates of clinical outcomes. Nonetheless, large RCTs did not support the use of routine TA in patients undergoing pPCI. Of interest, a recently published meta-analysis of 28 published studies by Bianchini et al. attempted to provide some insights into variables that were associated with an improvement in left-ventricle function in response to TA [54]. This included total ischemic time, left anterior descending artery (LAD) involvement, and TA technique [54]. Therefore, it is important to understand the limitations within these recent RCTs, which may explain this disconnect and may provide a platform to design future studies that could address the use of TA in patients presenting with STEMI.

Firstly, there was a lack of standard technical steps to guide TA during pPCI. Thrombectomy was used by crossing the thrombotic lesion and attempted to retrieve the thrombus by applying negative pressure during the pullback manoeuvre. This does not only reduce the efficacy of TA but also increases the risk of distal embolization by pushing the thrombus distally. Moreover, the volume of vacuum syringes was not standardized, and frequently, a single syringe was only used during thrombectomy. Furthermore, deep intubation of the guiding catheter was not recommended. This may, in theory, reduce the risk of thrombus dislodgement, causing peripheral embolization and stroke [41]. Overall, there is no consensus on the optimal use of TA in patients presenting with STEMI.

**Figure 1:**
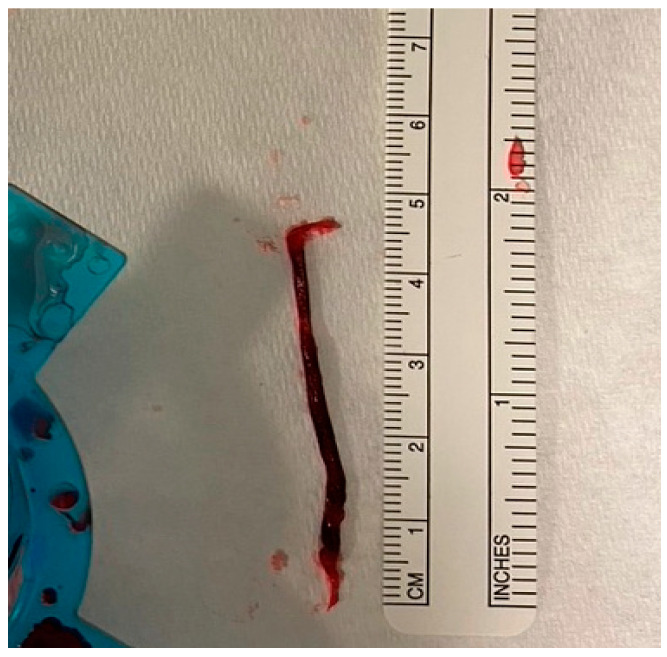
Large thrombus aspirated with manual TA during pPCI for anterior STEMI.

Secondly, large RCTs included all-comers of AMI cases and tested the benefits of routinely using TA in patients presenting with acute MI. Those patients have variable degrees of thrombus burden, and the purpose of thrombectomy is to extract the clot from the infarct site. Therefore, the rationale behind the use of TA in patients with a small thrombus burden is mechanistically not valid. On the other hand, thrombectomy should be considered in patients who have a large thrombus burden. This selective approach may allow a better assessment of the role of thrombectomy and whether it reduces cardiovascular risk. More importantly, the use of TA in patients with a small thrombus burden may dilute the result and mask any potential benefits of using TA in patients presenting with STEMI. In fact, an individual patient-level meta-analysis of the three large thrombectomy studies (TOTAL, TASTE, and TAPAS) showed that TA was associated with a small reduction in cardiovascular death in the high-thrombus-burden patient subgroup (TIMI thrombus grade ≥ 3) (2.5% vs. 3.1%; HR, 0.80; [95% CI, 0.65–0.98]; *p* = 0.03) [29] (Figure 1 illustrates an example of a large extracted thrombus). This finding was not present in patients with a low thrombus burden or in the whole cohort, highlighting the importance of a selective approach when applying TA in patients presenting with STEMI.

Thirdly, the currently used thrombectomy catheters are not very effective in retrieving thrombi. Manual thrombectomy catheters such as Export (Medtronic, crossing profile 0.068 in), Eliminate (Terumo Medical Corporation, Tokyo, Japan, crossing profile 0.068 in), OXT (Vascular Solutions, Minneapolis, MN, USA crossing profile 0.067 in), and Pronto (Vascular Solution, crossing profile 0.065 in) share the same concept of applying a vacuum at the proximal end in order to extract the thrombus from the plaque rupture site [44]. In fact, angiographic and imaging data suggest a large residual thrombus burden following the use of currently available thrombectomy catheters. The ineffective removal of a thrombus with a large residual thrombus burden (rTB) increases the risk of distal embolisation, NRP, and poor clinical outcomes [31,55]. Data from the TOTAL trial showed that a large rTB, as quantified by TIMI thrombus grade, was present in one third of patients who underwent TA [55]. Importantly, there was more than an 80% increase in the risk of the primary endpoint and cardiovascular death in patients with a large compared to small rTB following TA. Moreover, the risk of cardiogenic shock was doubled in this subgroup [55]. Similarly, the OCT sub-study from TOTAL, which included 214 STEMI patients, did not reveal any reduction in pre-stent thrombus burden with the adjunctive use of TA compared to PCI alone (2.36%, [95% CI, 1.73–3.22] vs. 2.88% [95% CI, 2.12–3.90], respectively, *p* = 0.373) [56].

To overcome the presence of a large thrombus burden, the ‘mother-in-child’ thrombectomy technique was developed to reduce the burden of thrombus in patients presenting with AMI. This technique is performed using a 5-French ‘Heartrail’ catheter (Terumo Medical) inside 6-French guiding system. In a small study of 13 patients, this technique reduced angiographic thrombus burden and improved coronary flow in 85% of patients.

## 5. Emerging Thrombectomy Technologies

**Figure 2:**
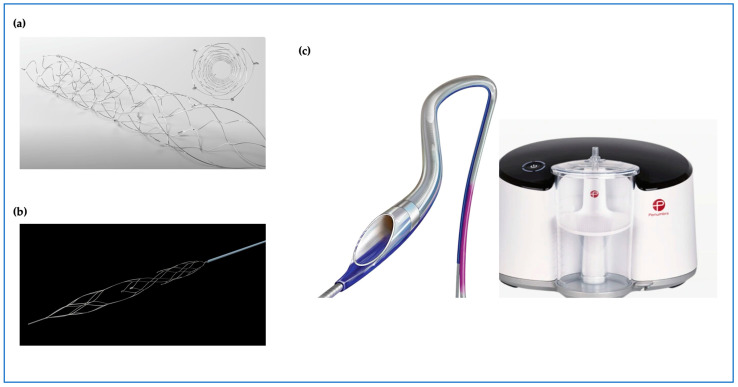
Emerging thrombectomy tool technologies with new designs; (**a**) **Solitaire™** device stent retriever (Medtronic). (**b**) **enVast^TM^** stent retriever (Vesalio). (**c**) **CAT RX** powered by Penumbra Engine.

Given the above inherent limitations in the design of thrombectomy catheters alongside their use in all-comer AMI patients, new technologies were developed to overcome these limitations, aiming to improve their efficacy in reducing thrombus burden and possibly clinical outcomes (Figure 2). Nonetheless, these tools had to be initially assessed to ensure safety during pPCI.

Mechanical thrombectomy using the Indigo CAT RX Aspiration System (Penumbra Inc., Alameda, CA, USA) was designed to provide continuous aspiration using a larger aspiration port and lumen (compared to manual aspiration catheters) and works with a Penumbra aspiration pump (Figure 2) [57]. The CAT RX is intended to provide a higher and more sustained vacuum to retrieve larger thrombus burdens and is currently used to treat ischemic stroke during neurovascular intervention. The CHEETAH Study (Sustained Mechanical Aspiration Thrombectomy for High Thrombus Burden Coronary Vessel Occlusion) investigated the safety of continuous mechanical thrombectomy prior to PCI in TIMI-thrombus-grade-4 and -5 AMI patients [57]. This single-arm registry enrolled 400 patients between August 2019 and December 2020 across 25 hospitals in the United States. It demonstrated the feasibility and safety of sustained mechanical thrombectomy in selected patients with a large thrombus burden. This was reflected in the low adverse CV event rate in this study with the primary endpoint of cardiovascular death, recurrent MI, cardiogenic shock, and NYHA IV heart failure of 3.60% [95% CI, 2.0–6.0%]. Additionally, the study reported a significant rate of final MBG grade 3 in 99.75% at the end of the procedure [57]. A previous meta-analysis reported increased mortality associated with mechanical thrombectomy [58]. It is important to highlight that the CAT RX catheter was not part of this analysis [58]. This meta-analysis referenced an ‘old’ technology whereby saline jets or rotating catheters were used to break up the thrombus prior to aspiration [58]. Importantly, there are no previous RCTs supporting the use of mechanical thrombectomy, and therefore, their efficacy as well as safety remain to be determined.

More recently, Spirito et al. reported the results of the first in-human use of the EnVast stent retrieval catheter (previously known as NeVa device) [59]. The authors included 61 patients presenting with AMI and a large thrombus burden and reported excellent safety data, with a single patient suffering from side-branch embolization. This phenomenon was subsequently overcome using the vacuum-assisted aspiration technique. Another side effect was reversible coronary spasm in 23% of patients, but there were no reports of coronary dissection or perforation. The results of EnVast on its own (prior to stenting) were excellent, with only 10% of patients having TIMI flow < 3 and almost one quarter having MBG < 2 [59]. EnVast received CE marking for use in AMI in 2019 [60].

Other stent retrieval technologies are also available for use in AMI patients. Few cases have been reported in the literature highlighting the benefits of using the nitinol stent-based self-expanding device (Solitaire, Medtronic) to retrieve a refractory thrombus from ectatic large coronary arteries [61,62]. The RETRIEVE AMI is a pilot RCT designed to assess the safety and efficacy of stent retrieval technology using Solitaire compared to conventional PCI or manual TA in patients with a large thrombus burden [63]. The primary endpoint will be the change in thrombus burden following thrombectomy and will be quantified using OCT. The study is planned to complete recruitment in 2024.

Overall, the safety profile of existing and emerging thrombectomy catheters is excellent. Given that these tools are delivered using a monorail system, there has not been any signal to suggest an increased risk of coronary dissection or perforation. Side-branch embolization has been reported with the use of stent retrieval catheters, but with increased experience and the use of a guide extension catheter, this risk could be minimized. Stroke has been reported as a potential side effect associated with existing thrombectomy catheters. This has not been consistent across all studies and is likely to reflect the used technique when deploying thrombectomy.

## 6. Conclusions

Thrombus burden is recognized to be a risk marker for patients presenting with AMI. Previous thrombectomy catheters have failed to show significant clinical benefits when tested in large RCTs. This may be related to the design of these studies or the used thrombectomy tools. Emerging thrombectomy technologies may provide more insights into the role of thrombus aspiration in patients presenting with AMI [64]. They could address the question as to whether decreasing thrombus burden is directly linked to reducing adverse clinical outcomes.

## Figures and Tables

**Table 1:** Thrombus burden classification [28].

Thrombus Grade	Definition
Grade 0	No thrombus.
Grade 1	Possible thrombus.
Grade 2	Small (greatest dimension ≤ ½ vessel diameter (VD)).
Grade 3	Moderate (>½ but <2VD).
Grade 4	Large (≥2VD).
Grade 5	Unable to assess TB due to vessel occlusion.

**Table 2:** Brief summary of previous manual thrombus aspiration trials *.

RCT and Date	Total Sample Size	TA Device/Catheter	Findings
REMEDIA (2005)	100	Diver CE Catheter	TA improves MBG (≥2) and STR (68.0% and 44.9% vs. 58.0% and 36.7%, respectively) with OR 2.6 ([95% CI, 1.2 to 5.9]; *p* = 0.020) and OR 2.4 ([95% CI, 1.1 to 5.3]; *p* = 0.034), respectively. TA is feasible and improves the angiographic and ECG criteria of myocardial reperfusion compared to PCI alone [36].
EXPIRA (2009)	175	Export Catheter	TA improved MBG (≥2) and STR (88% vs. 60%; *p* = 0.001; and 64% vs. 39%; *p* = 0.001). TA significantly reduced infarct size at 3 months, and lower cardiac death was observed at 9 months.
TAPAS (2008)	1071	Export Catheter	MBG 0 or 1 reported in 17.1% (TA with PCI) vs. 26.3% (PCI alone); (*p* < 0.001) [38]. Cardiac death at 1 year was 3.6% vs. 6.7% in TA vs. PCI alone (HR 1.93; [95% CI, 1.11–3.37]; *p* = 0.020) [39].
TASTE (2014)	7244	Multiple Catheters	Death from any cause occurred in 2.8% (TA group) vs. 3.0% (PCI group) (HR 0.94; [95% CI, 0.72 to 1.22; *p* = 0.63). Routine TA before PCI had no effect on 30-day mortality compared to PCI alone [44]. There was no reduction in death from any cause or composite of death, MI, or stent thrombosis at 1 year [46].
TOTAL (2015)	10,732	Export Catheter	Primary outcome occurred in 6.9% (TA group) vs 7.0% in PCI-only group (HR 0.99; [95% CI, 0.85–1.15; *p* = 0.86) with no reduction in CV death, recurrent MI, cardiogenic shock, or heart failure within 180 days [48]. Primary outcome at 1 year occurred in 8% in each group (HR 1.00 [95% CI, 0.87–1.15]; *p* = 0.99). CV death at 1 year was reported as 4% in each group (HR 0.93; [95% CI 0.76–1.14]; *p* = 0.48) [49].
INFUSE-AMI (2009)	452	Export Catheter	The intra-coronary Abciximab arm at 30 days showed a reduced infarct size (measured by cardiac MRI) compared to no Abciximab (median 15.1%; interquartile range [IQR] 6.8–22.7%; *n* = 181, vs. 17.9% [IQR] 10.3–25.4%]; *n* = 172; *p* = 0.03). TA vs. no TA showed no difference in infarct size at 30 days (median 17.0%; [IQR] 9.0–22.8%; *n* = 174, vs. 17.3% [IQR] 7.1–25.5%]; *n* = 179; *p* = 0.51) as well as no mortality difference at 1 year [50,51,52].

* CI: confidence interval, CV: cardiovascular, HR: hazard ratio, IQR: interquartile range, MBG: myocardial blush grade; MRI: magnetic resonance imaging, OR: odds ratio, RCT, randomised controlled trial, STR: ST-segment resolution, TA: thrombus aspiration; REMEDIA, Randomised Evaluation of the Effect of Mechanical Reduction of Distal Embolisation by Thrombus-Aspiration in Primary and Rescue Angioplasty; EXPIRA, Thrombectomy With Export Catheter in Infarct-Related Artery During pPCI; TAPAS, Thrombus Aspiration During Percutaneous Coronary Intervention in Acute Myocardial Infarction study; TASTE, Thrombus Aspiration in ST-Elevation Myocardial Infarction in Scandinavia trial; TOTAL, Trial of Routine Aspiration Thrombectomy with PCI versus PCI Alone; INFUSE-AMI, Intracoronary Abciximab and Aspiration Thrombectomy in Patients With Large Anterior Myocardial Infarction.

## Data Availability

Not applicable.
